# Single-Particle Photothermal Microscopy Using On-Chip Silicon Nitride Microring Resonators

**DOI:** 10.1021/acs.jpca.5c06077

**Published:** 2025-12-24

**Authors:** Yulia Podorova, Cecilia H. Vollbrecht, Samantha J. Evans, Hannah Rarick, Arnab Manna, Arka Majumdar, Randall H. Goldsmith

**Affiliations:** † Department of Chemistry, 201643 University of Wisconsin-Madison, 1101 University Ave, Madison, Wisconsin 53706, United States; ‡ Department of Electrical & Computer Engineering, 7284 University of Washington, Seattle, Washington 98195, United States

## Abstract

Optical microresonators confine light in time and space, enabling high sensitivity for the detection of small thermal perturbations. We utilized these properties and investigated on-chip integrated silicon nitride microring resonators (MRRs) for single-particle photothermal microscopy. We calibrate the technique using individual nonphotoluminescent carbon nanotubes and determine values for absorption cross-section per atom that agree with literature values. Finite-element simulations are performed to track all relevant thermal gradients. The combination of experiment and simulation allows for discussion of the benefits and drawbacks of the planar MRR geometry for single-particle microscopy.

## Graphical abstract

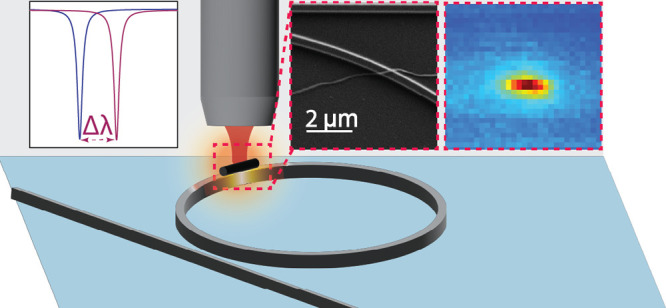

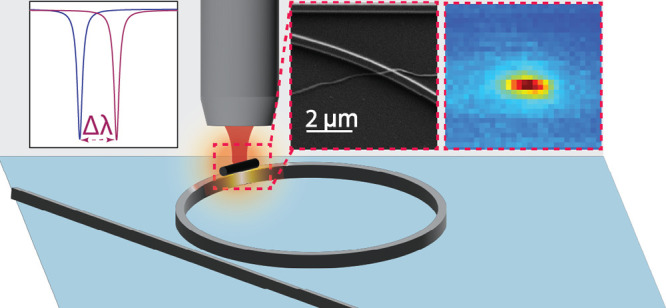

## Introduction

Optical whispering-gallery mode (WGM) microresonators confine light via total internal reflection in a closed loop geometry. Upon constructive interference after a round trip, light within the WGM microresonator builds up intensity, leading to high-quality factor (*Q*-factor) resonances. Consequently, optical microresonators have become a promising platform for many photonic applications. They are widely used as spectral filters/switches,^1^ optical delay lines,^2–4^ frequency comb generators,^5,6^ external-cavity frequency references for diode lasers,^7^ and integrated laser cavities when coupled to a gain medium.^8^ Microresonators are also highly sensitive to their local microenvironment,^9^ and have consequently been employed as label-free sensors for various targets like viruses, nucleic acids, proteins, lipids, and more.^10–30^

However, small changes in temperature at a microresonator surface cause changes in the effective refractive index experienced by the optical mode, which results in a shift of the resonance wavelength. This behavior is a detriment to many applications in photonics but opens up opportunities as a highly sensitive thermometer that can be leveraged for sensitive photothermal microscopy and spectroscopy.^29–50^ Single-particle photothermal microscopy has many applications in physical chemistry because it enables the interrogation of nonemissive particles and molecules. Particularly high sensitivity to controlled thermal fluxes can be achieved with microresonators in a two-beam geometry (Figure 1):^31^ A probe beam, a continuous-wave narrow-band wavelength-tunable laser, is monitoring the WGM resonance, while a pump beam, a free-space beam tightly focused on the resonator surface, is used to drive excitations in the particle of interest. When the pump beam excites an absorber on the resonator surface, such as a single particle or molecule, the absorbed energy is dissipated as heat into its local environment. The heat at the microresonator surface causes a change in the effective refractive index, which results in a shift in the resonance, as detected by the probe beam. This geometry has been used for a variety of applications, including exploring hybrid photonic-plasmonic systems,^51–55^ revealing underlying electronic structure and ordering in conducting polymers^56,57^ and following chemical reaction dynamics on single nanorods.^58^

**Figure 1:**
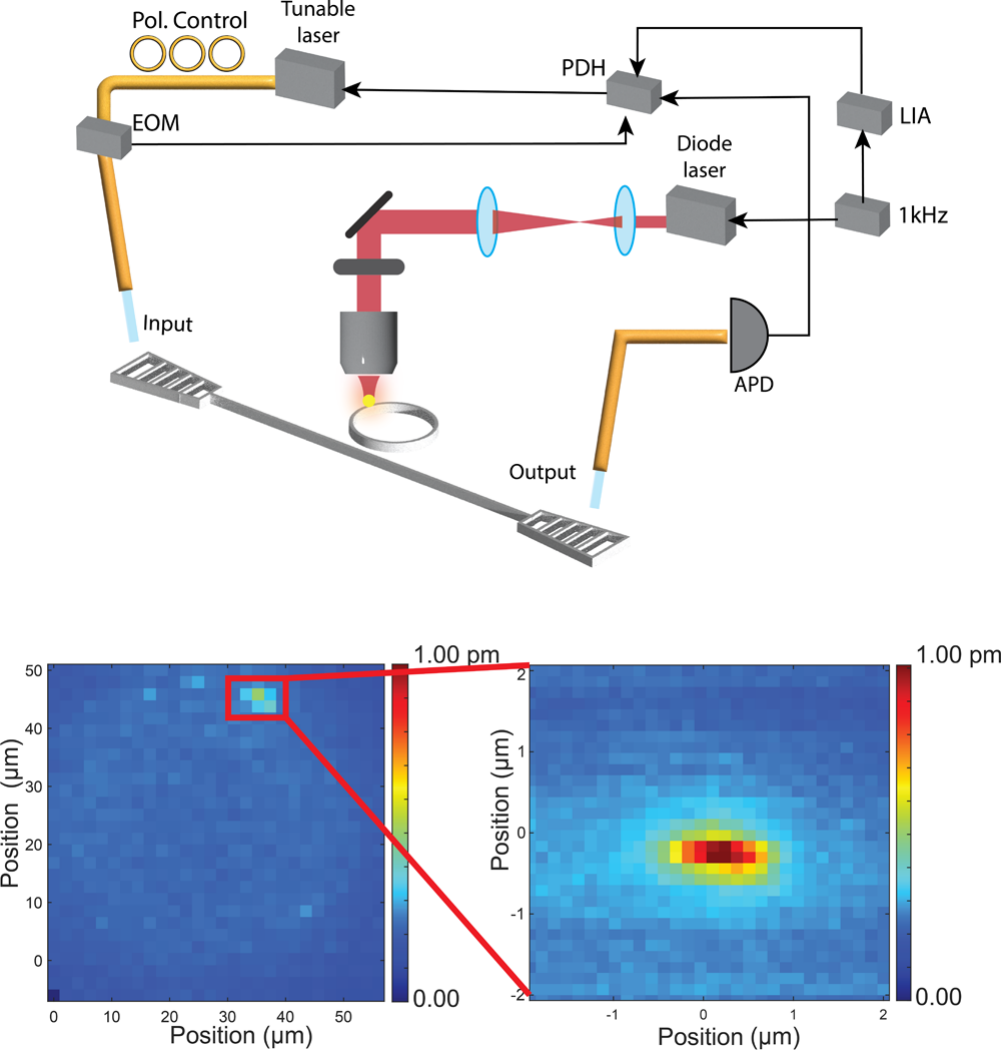
(Top) Schematic of the optical path of the pump laser and the fiber-coupled probe laser. Abbreviations: PDH = Pound-Denver-Hall locking; LIA = lock-in amplifier; EOM = electro-optical modulator; and APD = avalanche photodiode detector. (Bottom left) Full photothermal map of a MRR with MWCNTs deposited. (Bottom right) Close-up photothermal map of a MWCNT on a MRR.

While multiple geometries of microresonators^52,57–61^ such as microtoroids^62^ or microbubbles^63^ have been used for photothermal spectroscopy and microscopy, the need for at least one fabrication step to be stochastic or individually handled proves to be a major challenge for widespread adoption: each microtoroid required a reflow step to achieve the desired smoothed torus geometry;^9^ each microbubble is fabricated one at a time using two counter-propagating CO_2_ laser beams focused on the capillary.^64^ In both cases, the stochasticity of these steps results in considerable heterogeneity in the geometry of the microresonator. Even in chip-scale reflows of all-glass microtoroids, considerable toroid-to-toroid heterogeneity in dimensions persists.^60^ Here, we evaluate the performance of planar microring resonators (MRRs), which can be produced uniformly at a chip-scale, as ultrasensitive thermometers for single-particle microscopy. As a model system, we use multiwall carbon nanotubes (MWCNTs) as single-particle nanoabsorbers.

The geometry of MRRs could bring multiple additional benefits as compared to microtoroids and microbubbles, which are nonplanar structures. Microtoroids and microbubbles required use of tapered optical fibers, which are fragile, sensitive to the environment (airflow, dust, mechanical vibrations), and require precise nanopositioning.^9,12,62^ The complex geometry can lead to imaging artifacts when used in a backside imaging configuration.^59^ On the other hand, complex geometries also provide opportunities for exploring target materials at a range of orientations with respect to the optical axis.^57^ In contrast, the planar geometry of MRRs allows fabrication of on-chip integrated waveguides which can be accessed via grating couplers,^65^ Figure 2, while samples could be deposited and imaged analogously to fully far-field techniques that do not require a nearby microresonator.

**Figure 2:**
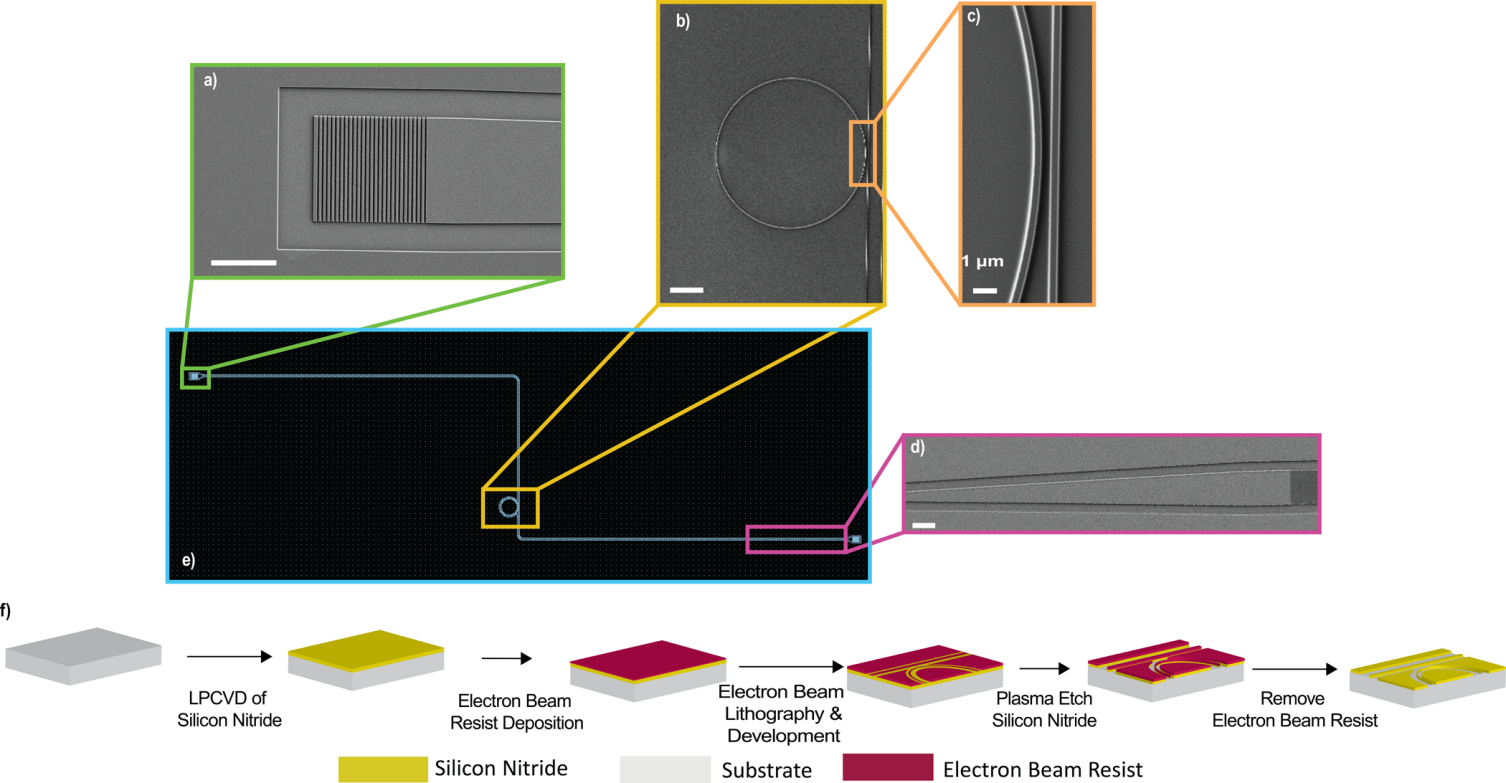
MRR design and fabrication: (a) GC, (b) ring resonator, (c) coupling region, (d) tapering region, (e) full design, and (f) schematic of the fabrication process flow.

Two key figures of merits, the *Q-*factor and mode volume (*V*),^66^ need to be considered in designing MRRs for single-particle photothermal spectroscopy. Higher *Q*-factors lead to sensitivity to smaller changes in the resonant wavelength and thus to smaller thermal fluxes. Losses associated with internal absorption, surface scattering, or radiative loss of the probe beam all contribute to lower *Q*-factors. High *Q*-factors in MRRs (>10^7^) have been achieved by using delocalized resonators,^67–69^ increasing the ring diameter,^70^ adding a cladding,^71^ using telecom wavelengths,^72^ adding an annealing step during fabrication,^73,74^ or chemical mechanical polishing.^72,75^ On the other hand, the mode volume quantifies the spatial confinement of the electromagnetic field within the microresonator. Smaller mode volumes increase microresonator sensitivity to local environmental perturbations, such as those caused by the heat dissipated by a single particle due to the greater overlap between the optical mode and the increased temperature profile. There is a fundamental trade-off here, as larger *Q* values can be achieved by making the microresonators larger but at the cost of the photothermal signal being diluted in the large mode volume, while even smaller mode volumes can be reached by decreasing the microresonator radius, but such modification leads to higher bending losses and therefore lower *Q*-factors.^76^ For single-particle microscopy, the highest possible ratio of *Q*/*V* is desirable to maximize the responsivity to the photothermal signal. Other factors such as background absorption of the medium (discussed below), modulation frequency, and time-stability of the resonator-waveguide can also influence the ultimate sensitivity.^52,58,60^

The optimal material choice must also be considered. For microtoroids, even though the mode propagates in silica, the silicon support pillar contributes to photothermal background, even when it is thinned.^31,77^ All-glass microtoroids with chip-scale fabrication have been reported but even these devices possess a high photothermal background, likely due to residual unoxidized silicon.^60^ Silicon nitride (Si_3_N_4_) can be deposited with a range of silicon to nitrogen stoichiometries depending on the deposition parameters and the ratio between the two greatly affects properties such as the refractive index and internal material stress. Higher stress, stoichiometric silicon nitride exhibits much less internal absorption in visible wavelengths, making it the better choice in our experiment.^78^ As compared to silica, the higher index of silicon nitride allows fabrication of smaller MRRs while still minimizing radiative losses. Silicon nitride has a higher thermo-optical coefficient, exhibits low losses, and is transparent at a wide range of wavelengths, from mid-infrared to as low as 500 nm, which makes it an excellent candidate for thermo-optical detection applications.^79^ A table showing comparison of refractive indices, thermo-optical coefficient, and thermal conductivity for silicon, silica, and silicon nitride is presented in the SI. The MRRs we present here have a height of 275 nm and width of 450 nm, which already significantly decreases the mode volume compared to microtoroids.

## Methods

### Device Design

Each photonic circuit includes a pair of grating couplers (GCs) for input and output coupling, a 41 mm long s-shaped waveguide with 1000 μm tapering region, and a 30, 45, or 60 μm diameter MRR, Figure 2. Use of the s-shaped waveguide makes it easier to distinguish out-coupled photons from photons that may have been guided by other interfaces. The coupling gap between the ring and the waveguide is measured to be 380 nm, which allows light from the waveguide to efficiently evanescently couple into the MRR without substantially increasing loss. The GCs are Bragg gratings with a series of ridges and trenches. A benefit of GCs is that they are fully complementary metal-oxide semiconductor (CMOS) compatible and can be fabricated simultaneously with the rest of the device allowing for flexibility on-chip design.^80^ One drawback is that GCs are designed to work at a specific wavelength and, therefore, have increased losses when tuning over wide wavelength ranges. GCs were designed based on comparisons to previous designs.^81–83^ The GCs were designed for a 10° input angle for 777–780 nm wavelength. The GCs are 15 × 15 μm to match a single-mode fiber. The final design had a period of 590 nm. The ridges have a width of 410 nm and the trenches were 180 nm wide. The silicon nitride in the area around the microring is fully etched away to minimize sources of photothermal background. The full details of GCs design can be found in the SI.

### Fabrication

The fabrication scheme is summarized in Figure 2f. Full fabrication description can be found in the SI. In brief, four-inch silicon wafers with 2 μm of thermal silicon oxide were diced into one-by-two-inch pieces. After dicing, the pieces underwent Piranha, RCA SC-1, and RCA SC-2 cleaning procedures. Silicon nitride was then deposited on clean pieces using low pressure chemical vapor deposition with dichlorosilane and ammonia gases. Photoresist ZEP520A was spun on each piece; the resonator pattern was then defined on the pieces using electron beam lithography. The pieces were then developed using xylenes and IPA. The pattern was transferred to silicon nitride via plasma etch. After etching, any remaining electron beam resist is removed by soaking the pieces in 1165 remover. The pieces are then rinsed with IPA and dried with nitrogen.

### Particle Deposition

Microring resonator wafers were sonicated in IPA for 15 min, dried with nitrogen, and plasma cleaned for another 15 min. The wafer was then placed on a hot plate at 40 °C. MWCNTs (Sigma-Aldrich, >90% carbon basis, used without further purification) were dissolved in *N*-methyl-2-pyrrolidone (Sigma-Aldrich, ACS reagent, ≥99.0%) at a concentration of 1.25 μg/mL, sonicated at 35 °C, and were drop-cast onto the resonator wafers. During drop-casting, a single 2 μL drop of solution is used, and multiple MRRs are deposited upon at once, allowing for chip-scale particle deposition. Scanning electron microscopy (SEM) images were taken before photothermal experiments to confirm the position of the particles and after to ensure particles were not damaged during the imaging process.

### Optical Characterization of MRRs

A fiber-coupled narrow line width external-cavity tunable diode laser (Newport, TDL-6712, 761–781 nm) provides the probe beam. The laser output is connected to polarization paddles followed by an electrooptic modulator (EOM, Jenoptik), followed by a single-mode patch cable (Thorlabs, P3-780Y-FC-2) cleaved using a manual fiber cleaver (TM27 High Precision Fiber Optic Cleaver). The cleaved end of the fiber is aligned to the input GC. Another cleaved patch cord is aligned to the output GC, and the light is transmitted to an avalanche photodetector (Thorlabs, APD430A).

As mentioned above, increasing the *Q*-factor and the overlap between mode volume and the temperature profile of the particle will in turn elevate photothermal sensitivity (Figure S2). For our photothermal experiment, we used MRRs of diameter of 45 μm, which provides a small enough mode volume without introducing significant radiative losses and maintaining *Q*-factors of 6 × 10^4^.

The probe laser is actively locked to the microring resonance via the Pound-Drever-Hall (PDH) locking technique, and the error signal is used to monitor any changes in the microring resonance wavelength.^19,52,84,85^ PDH feedback is applied to the tunable laser using a high-speed servo controller (Newport LB1005).^52^

### Photothermal Experiments

A fixed wavelength diode laser (Blue Sky Research, 640 nm) is used to optically pump the MRRs and target nano-objects. The excitation beam passes through a λ/2-plate, which is mounted on an automated rotation stage to allow for tuning the polarization of the pump with respect to the target object and measure polarization dependent data. The excitation beam is delivered by a 60*x*/0.95NA objective, resulting in a near-diffraction-limited spot 360 nm diameter. The pump beam is amplitude modulated at 1.1 kHz. The resulting photothermal signal was extracted using a lock-in amplifier (Ametek 7265).^52,58^

Previously, galvo metric mirrors were used to control the position of the excitation beam;^58^ however, this scheme limits the size of the scan area. Instead, a stage was designed to hold the MRR chip and the spliced patch cords, and the position of the whole system is moved together relative to the excitation spot with nano positioners (Attocube). Mechanical perturbations are a concern due to a minimum required settling time of the nano positioners; therefore, to collect spatial data first, a waiting time of 0.5 s was allowed after shifting positions, and photothermal data are collected while the system is not in motion.

### Simulations

Finite-element simulations (COMSOL) are used to model optical modes inside the MRR, thermal properties of the MRR, and the wafer upon optical excitation and heat transfer from a single particle. Both time-dependent and steady-state results were evaluated. The optical mode, Figure 3, was modeled using the “Axisymmetric Cavity Resonator” guide in COMSOL Multiphysics.^86^ The dimensions, materials, and resonance wavelength were adjusted appropriately, and more information on simulation parameters can be found in the SI. The resulting electromagnetic field was later used to evaluate resonance shift.

**Figure 3:**
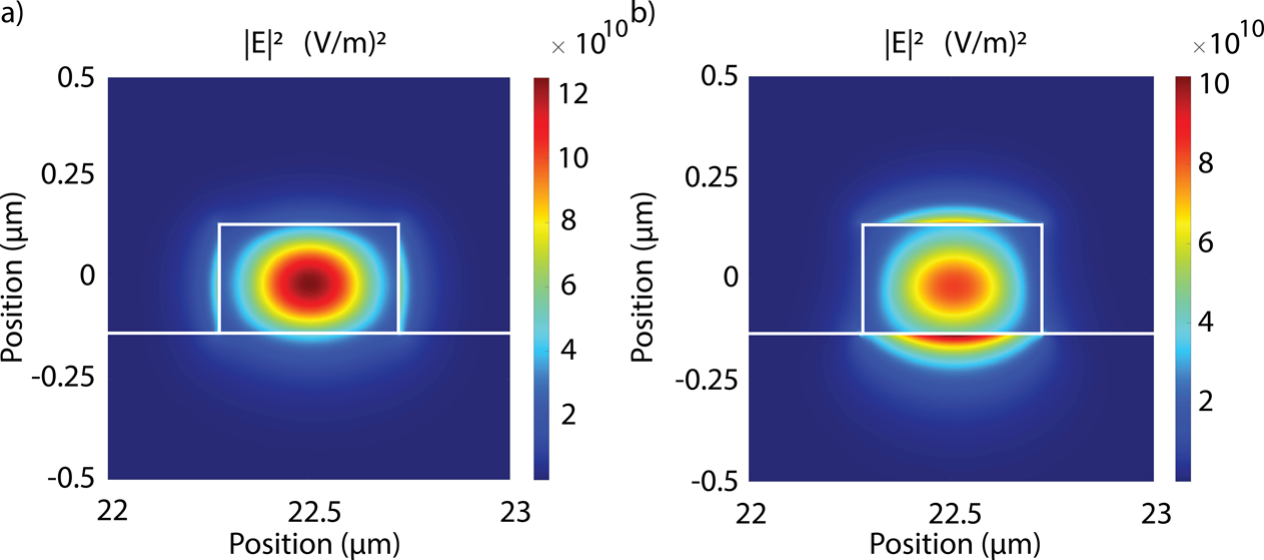
Optical mode |*E*|^2^ (V/m)^2^ of the cross-section of MRR: (a) transverse electric mode; (b) transverse magnetic mode.

To evaluate heating from the pump laser, the “Modeling the Pulsed Laser Heating of Semitransparent Materials” blog post was used as a guide.^87^ All three layers: Si_3_N_4_, SiO_2_, and Si were incorporated with the appropriate dimensions and material properties. More details can be found in the SI.

To evaluate heating from single-particle sources, the model included a full MRR while multiwalled carbon nanotubes (MWCNTs) were modeled as cylinders of the same size and position as those measured via scanning electron microscopy (SEM) and were set as heat sources. The bounding surfaces of the silicon platform and surrounding air were kept at constant temperature. Steady-state solutions to the thermal simulation were obtained, with software-defined extrafine mesh used for the MRRs. The electromagnetic field from the optical mode simulation was imported as a function of the thermal simulation. Azimuthal revolution was used to convert the 2D simulation into a 3D simulation. The resonance shift was determined by calculating by using a software-defined volume integration function of the azimuthal overlap of the optical mode and the thermal profile of the MRR. The dissipated thermal power from the particles was estimated and iterated to match the resonance shift seen in experiment.

## Results and Discussion

Photothermal images were acquired by scanning the sample with respect to the pump beam, Figure 1. Wide-area maps (50 × 50 μm) were taken at low resolution (2 μm/pixel). These maps are used as a fast scan to locate particles. Figure 4 shows the results of closely examining two MWCNTs located on the same MRR at two different locations. Small area maps were taken with higher resolution, 500 nm/pixel, (Figure 4a) and were accompanied by SEM images (Figure 4b). Thus, Figure 4 shows that planar silicon nitride MRRs can be used for single-particle photothermal microscopy. Photothermal signals are well-correlated with MWCNTs observed in SEM micrographs, as shown via spatially overlapped images in Figure S3.

**Figure 4:**
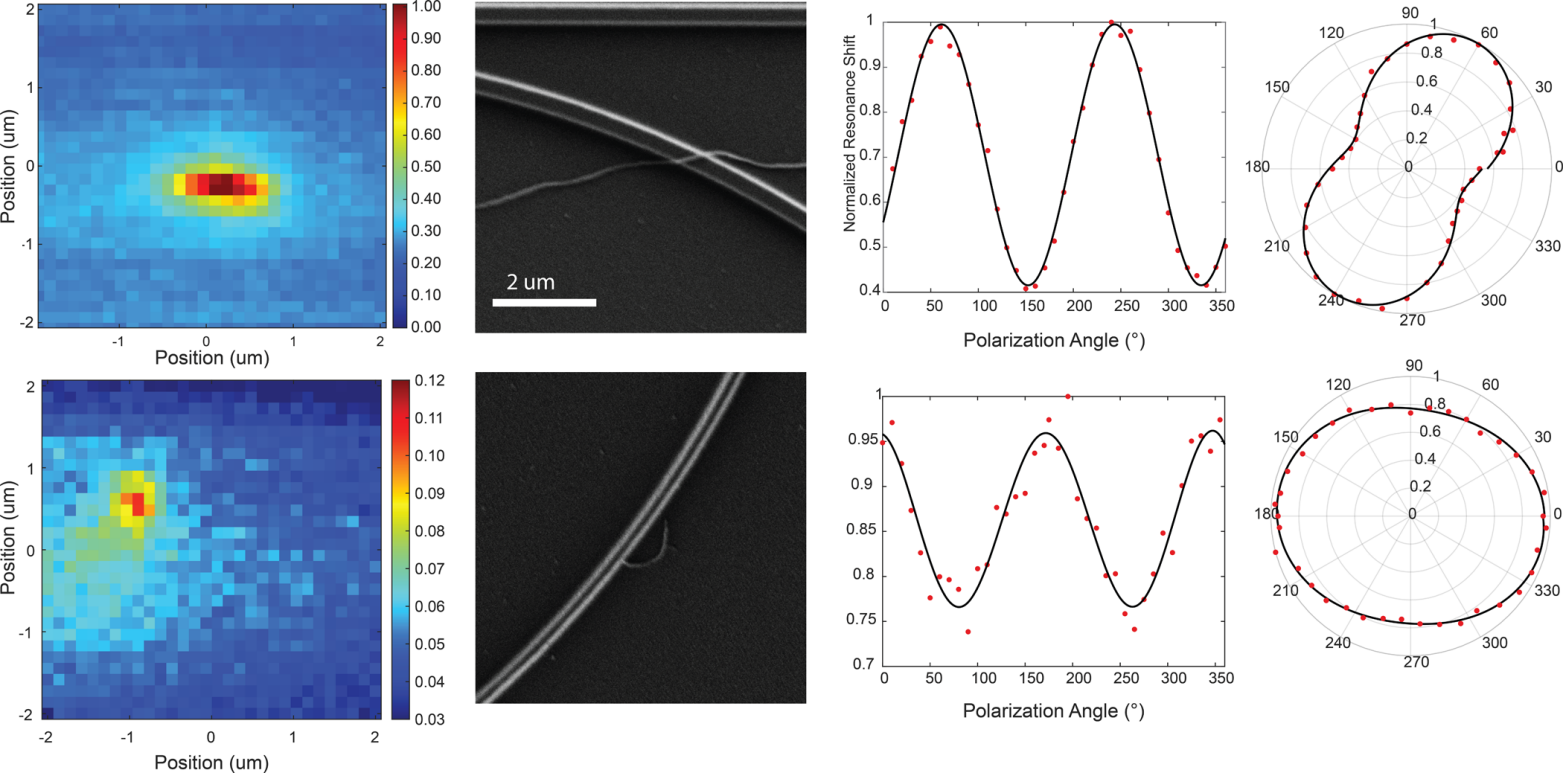
Photothermal characterization of two different MWCNTs. (a) Photothermal maps collected at 500 nm/pixel at 635 nm excitation wavelength and 500 μW optical power. (b) Scanning electron micrographs. (c) Polarization dependence of photothermal signal. (d) Polarization dependence plotted in polar coordinates.

The polarization dependence was also quantified, Figure 4c,d. SEM images were taken before and after photothermal mapping to identify locations, size, and shape of MWCNTs, and to confirm that little photodegradation had occurred. The SEM images are oriented true to the particles’ positioning on the ring. The polarization trends qualitatively match the orientations of the particles. For particle I, the polarization extinction ratio was measured to be 2.1:1, while for particle II, it is 1.3:1. After closer examination of SEM images, we determined that particle II is actually a combination of two different particles, which leads to the lower polarization extinction ratio. The lack of complete extinction is consistent with previous measurements of both MWCNTs, where the curvature of the MWCNT reduces the polarization asymmetry,^52^ and single-walled nanotubes, where symmetry breaking was observed due to interactions with the substrate.^88–90^

The absorption cross- section (σ_abs_) per atom (Table 1) was calculated using the input optical power (*P*_optical_) and dissipated heat power simulated in COMSOL (*P*_thermal_) using the following equation:^31^

$$\sigma_{\mathrm{abs}}=\frac{P_{\mathrm{thermal}}}{P_{\mathrm{optical}}}\times \frac{1}{\left(1-\varphi_{\mathrm{lumin}}\right)}\times \left[\pi {\left(\frac{w_{0}}{2}\right)}^{2}\right]\times \frac{M}{\pi {\left(\frac{d}{2}\right)}^{2}L\rho N_{a}}\times \beta$$

where φ_lumin_ is the quantum yield for luminescence (0 for MWCNT), *w*_0_ is the 1/e^2^ beam diameter (780 nm), *d* and *L* are MWCNT diameter and length, respectively, ρ is the density (1.75 g/cm^3^),^91^ *N*_a_ is Avogadro’s number, *M* is the molar mass (12.01 g/mol carbon), and β is the fraction of atoms in the nanotube excited by the pump beam. β is calculated as a 2D integral of a product of two functions: the number of atoms in the nanotube and the Gaussian shape of the excitation beam, divided by the total number of atoms in the nanotube.

**Table 1:** Parameters for Photothermal Measurement of MWCNTs

	length (μm)	diameter (μm)	resonance shift (pm)	power dissipated (μW)	carbon fraction absorbing, β (%)	abs cross-section (1 × 10^–18^ cm^2^/ atom)	polarization extinction ratio
**I**	6.65	0.105	1.01	93	11.1	2.24	2.1:1
**II**	1.51	0.071	0.095	14	27.7	0.902	1.3:1

Particle dimensions, resonance shift, and polarization excitation ratio are measured directly using SEM or photothermal microscopy. The dissipated heat power is estimated by using finite-element simulation. The carbon fraction absorbing and the absorption cross-section are calculated by using a combination of measurements and simulation. The average absorption cross-section is determined to be 1.6 ± 0.5 × 10^–18^ cm^2^/C atom, which is comparable to previously reported MWCNT single-particle spectroscopy result of 2.3 ± 0.5 × 10^–18^ cm^2^/C atom using microtoroid resonators.^31^ MWCNTs of this size (>100 nm diameter) can be compared to bulk graphite due to the MWCNTs’ relatively gentle curvature. Reported per-atom cross-sections of graphite range over 2.5–2.8 × 10^–18^ cm^2^/C atom.^92–95^ Literature values of 1–3 × 10^–17^ cm^2^/C atom are reported for single-walled carbon nanotubes^88,96–98^ but with a strong dependence on wavelength and tube chirality. MWCNTs, as multilayer structures, are expected to have optical properties more comparable to those of graphite, as observed.

The signal-to-noise ratio (SNR) is 282 ± 7, and the signal-to-background ratio is 3.5 ± 0.1 (SBR, particle I). The SNR was calculated by taking the ratio of the lock-in amplifier output with the excitation beam centered over the particle and unblocked (signal) to the root-mean-square (RMS) value obtained when the beam was blocked. The SBR was calculated as a ratio of the average lock-in amplifier output when the pump beam is focused on a particle compared to an area of the MRR where no particles were present. A similar imaging scheme using microtoroids as the photonic platform showed somewhat a higher SNR (425) but a much higher SBR ratio (36).^52^ While in microtoroids, the main source of noise was attributed to the difficulty in maintaining constant coupling using tapered fibers;^52^ MRRs avoid this problem due to their planar geometry. However, the significantly lower *Q*-factors of the MRRs (6 × 10^4^ as compared to 2 × 10^7^) contribute to the lower SNR.

The low SBR is a major issue, particularly for future application to objects with much smaller absorption cross-section, such as single molecules. To explore the underlying causes for the low SBR, we performed experiments and simulations where no MWCNTs were added and the pump laser pumped only the intrinsic absorbers of the component materials. Finite-element simulations were performed in COMSOL to account for the thermal contributions of all the materials components on the background resonance shift. Figure 5 shows the data from these studies for clean MRRs in the form of resonance shift vs spatial position on MRRs of three diameters: 30, 45, and 60 μm. Although the MRRs are fabricated from silicon nitride and are optically transparent at 635 nm (the pump wavelength), they are fabricated on silicon wafers that strongly absorb visible pump wavelengths. Upon absorption of visible photons and rapid thermalization, some of the thermal energy will transfer to the silica layer and then to the nitride layer where a background resonance shift will be induced. To reduce that photothermal background, a relatively thick 2 μm layer of thermal silicon oxide was used between the nitride and silicon layers to act as a thermal insulator.^79^ Qualitative agreement is reached between simulation and experiment, suggesting that the simulation captures the key physics of the detection scheme.

**Figure 5:**
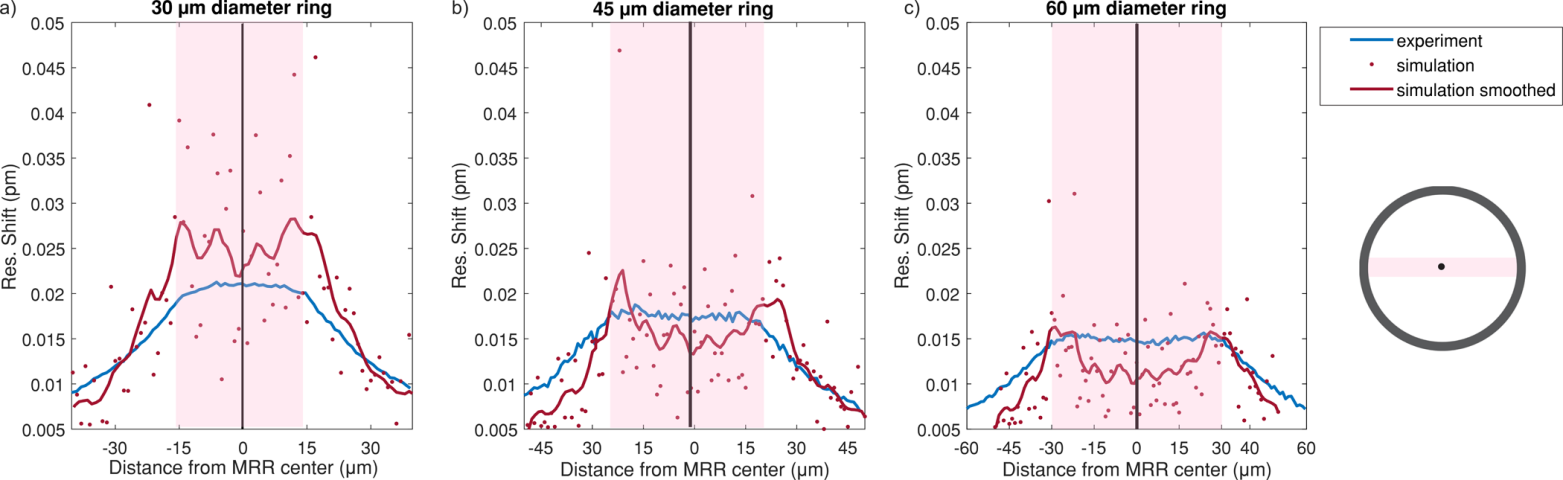
Background photothermal signal for (a) 30, (b) 45, and (c) 60 μm diameter MRRs, experimental data collected with 500 μW optical power depicted in blue traces, and simulated data depicted in red traces. The pink shaded regions indicate the area within the MRR diameter. The diagram on the right relates the *x*-axis to the dimensions of the MRRs.

Just as the platform is contributing to the high photothermal background, it also contributes to the loss of signal from single particles. Even though the heat from the silicon wafer can transfer up to the nitride MRR, the heat from the MWCNTs on the MRR can also travel down into the silicon wafer, reducing the magnitude of the photothermal signal. Once heat from the particle transfers to the MRR, it travels quickly through the thermally conductive (10 W m^–1^ K^–1^) silicon nitride,^99,100^ before settling into the more insulating SiO_2_ (1.4 W m^–1^ K^–1^) layer,^101^ and then dissipates into the very thermally conductive (140 W m^–1^ K^–1^)^102^ silicon wafer where it effectively leaves the system. To evaluate how the surroundings contribute to the loss of heat from the MWCNT, finite-element simulations were employed. A simulated MWCNT particle of 1 μm length and 0.1 μm diameter was modeled as a point source dissipating heat of 100 μW, comparable to size and dissipated thermal power report here and the previous MWCNT study.^31^ Several underlying geometries were simulated to explore the mechanism of signal transduction: (1) the design used in experiment: 45 μm diameter MRR on 2 μm layer of silicon oxide on 1000 μm layer of silicon; (2) 45 μm diameter MRR on 1002 μm layer of silicon oxide (no silicon); (3) 45 μm diameter MRR suspended in air (an unphysical geometry); (4) a silicon oxide microtoroid with major radius 20.5 μm and minor radius of 2.5 μm on a silicon pillar 5 μm diameter (skinny pillar); and (5) a silicon oxide microtoroid with major radius 20.5 μm and minor radius of 2.5 μm on a silicon pillar 20 μm diameter (standard pillar). The last two designs were chosen to directly compare to our previous geometry for single-particle photothermal microscopy.^77^

These results, shown in Table 2, show a strong dependence of signal strength on the substrate geometry. Removing the highly thermally conductive silicon, as has been achieved with microtoroids,^60^ is predicted to enhance the signal by a factor of 2.2. Completely eliminating the substrate, an experimentally difficult geometry to realize, results in a substantial further increase in the signal, illustrating how different platforms contribute to heat distribution in the system Figure S1. However, note that increasing the thermal isolation of the system will also reduce the maximum modulation rate, potentially introducing sources of technical noise. This trend is similar to the one observed with microtoroids on different size support pillars,^77^ where it was shown that use of a smaller size pillar reduces the heat capacity of the pillar leading to greater temperature increase in the mode-volume-carrying silica toroid, and thus elevated background. Still, for highly sensitive photothermal spectroscopy, some degree of thermal isolation of the microresonator is essential. Even though the MRRs offer a substantially smaller mode volume, their reduced thermal isolation causes a drop in signal magnitude for an equivalent thermal source of 3.3x (comparing Rows 1 and 5, a typical microtoroid experiment). The drop can be partially mitigated by a less conductive substrate. This drop, seemingly intrinsic to the planar geometry, along with the substantial drop in *Q*-factor, combined to reduce the potential of MRRs for single-particle spectroscopy.

**Table 2:** Predicted Heat Dissipated from a Single MWCNT Particle and the Corresponding Resonance Shifts for Different Platform Models^a^

model	average temperature change in the microresonator (K)	predicted optical resonance shift for λ_res_ = 780 nm (pm)	relative ratio to row 1
1	0.486	3.82	1:1
2	1.08	8.48	2.2:1
3	25.6	205	53:1
4	8.43	90.7 (predicted for λ_res_ = 1550 nm)	23:1
5	1.09	12.6 (predicted for λ_res_ = 1550 nm)	3.3:1

a See text for details.

Trends in photothermal sensitivity for microresonators of different geometries^77^ were also remarked upon by Su and co-workers.^103^ Here, the elevated sensitivity of a thermally decoupled microtoroid^77^ was used to partially explain claims to photothermally image objects with smaller absorption cross-sections, including single emissive quantum dots (QDs) of size 5 nm. However, we note that analysis analogous to what is presented here around Table 2 is not performed in ref 103. Rather, instead of empirically determining an absorption cross-section and comparing to an independent experimental value, as done above and in previous works^31,52^ with MWCNTs and gold nanorods, there is no independent, calibrated determination of the QD cross-section. Instead, detected nano-objects are assumed to be single QDs and a thermal sensitivity is then calculated. Until such a comparison is made, it is unclear whether such resolution is possible with microresonators.

More generally, a variety of single-particle photothermal readouts have appeared in the chemical instrumentation literature over the past 15 years, including several that have achieved single-molecule resolution^35,43^ or imaging of objects with comparable absorption cross-section.^57^ Some of these methods, such as photothermal heterodyne imaging (PHI)^35,38^ and force microscopy,^44–46^ only require the substrate of the system of interest to be transparent and derive sensitivity from sophisticated optical or scanning probe readouts. Other approaches such as use of ultrahigh-*Q* optical microresonators or atomically thin high-*Q* mechanical resonators entail simple pump geometries but require specialized substrates for readout.^43^ Though emphasis is justifiably placed on the high-*Q* values of these measurement systems for achieving high sensitivity, these systems also benefit from a degree of thermal isolation (in some cases, to a substantial degree). Use of planar MRRs reduces this benefit, and our analysis quantifies the thermal flows responsible.

## Conclusions

A planar microresonator, an MRR, was investigated for applications in single-particle photothermal microscopy. Individual nonemissive MWCNTs were imaged, and the polarization dependence and per-atom absorption cross-section were quantified. Importantly, the per-atom cross-section agrees with the literature values. The SNR and SBR were determined to be 282 and 3.5, respectively. A combination of simulations and experiments was used to unravel the role of sample geometry in determining the fate of heat underlying signal and background channels. Even as the planar geometry offers many advantages in terms of fabrication and photonic integration, the planar geometry is conclusively shown to mute signal while amplifying the background. Looking forward, fabricating MRRs on an all-transparent platform like quartz or sapphire can improve the SBR. Further fabrication efforts, like polishing or annealing Si_3_N_4_ to raise the *Q*-factor, can also increase the overall sensitivity of the platform.

## Supplementary Material



## Notes

Material properties; GC design; device fabrication; simulation details; thermal simulations of different platforms; optical characterization of MRRs; and photothermal mapping of MWCNTs (PDF)

## §

Department of Chemistry and Biochemistry, Kalamazoo College, 1200 Academy Street, Kalamazoo, Michigan 49006, United States

## Abbreviations

WGM: whispering-gallery mode
MRR: microring resonator
GC: grating coupler
MWCNT: multiwall carbon nanotubes
SEM: scanning electron microscope
PDH: Pound-Denver-Hall
LIA: lock-in amplifier
EOM: electro-optical modulator
APD: avalanche photodiode detector
SNR: signal-to-noise ratio
