# Supplementary material for: Single-Particle Photothermal Microscopy Using On-Chip Silicon Nitride Microring Resonators
Source: J Phys Chem A. 2025 Dec 24;130(1):298–307. doi: 10.1021/acs.jpca.5c06077 (PMC12794187; doi:10.1021/acs.jpca.5c06077)
Supplement: Supplementary file 1 [file jp5c06077_si_001.pdf]

Supporting Information for

Single-particle photothermal microscopy using on-chip silicon nitride microring resonators

Yulia Podorova [†‡], Cecilia H. Vollbrecht [†‖‡], Samantha J. Evans [†], Hannah Rarick [§], Arnab Manna [§], Arka Majumdar [§], Randall H. Goldsmith [†*]

[†]University of Wisconsin-Madison Department of Chemistry, 1101 University Ave, Madison, WI, US 53706

[‖] Current Address: Kalamazoo College, Department of Chemistry and Biochemistry, 1200 Academy Street, Kalamazoo, MI, US 49006

[§] University of Washington, Department of Electrical & Computer Engineering, Seattle, WA, US 98195

*Email: rhg@chem.wisc.edu

Table of Contents:

S2 Material properties

S2 Grating coupler design

S3 Device fabrication

S4 Simulation details

S5 Thermal Simulations of different platforms

S7 Optical characterization of MRRs

S8 Photothermal mapping of MWCNTs

Material properties

	Silicon	Silica	Silicon Nitride
**Refractive index[1-3]**	3.71	1.45	2.02
**Thermo-optic coefficient[4-6] (K^{-1})**	$1.86x10^{-4}$	$8.6x10^{-6}$	$2.45x10^{-5}$
**Thermal Conductivity[7-9] at 300K ($\frac{W}{m*K}$)**	156	1.11	25-36

Table S1. Optical and thermal properties of silicon, silica and silicon nitride

Grating coupler design

Grating couplers were designed based on comparisons to previous designs, calculations of expected peak wavelength, and simulations performed in Lumerical software to optimize transmission at a given input angle (10°) and wavelength (777-780nm). Calculations of expected maximum transmission wavelength were performed using the conditions for a Bragg grating, as shown in equation 1[10]:

$$\text{Grating Period} = \frac{m\lambda}{n_{eff} - n_1 sin\theta}$$

In this equation, m is an integer multiplier, lambda is the peak wavelength, theta is the angle of incidence of light on the grating, n_{eff} is the effective refractive index of the entire grating, and n_1 is the refractive index of the cladding. The following parameters were set during calculations and simulations to simplify fabrication. The etch depth was set as the depth of the nitride deposited so that all etching could be achieved in one step across the device.[11] The angle of incidence was set to 10° to prevent back reflection and match equipment used in the physical setup design. The cladding was assumed to be air since the MRR needed to be exposed to air to allow deposition of samples. Therefore, the ridges in the grating coupler were silicon nitride and

the troughs were air. The width of the ridges and period were allowed to vary. The area of the grating coupler was set to 15 µm x 15 µm to match a single-mode fiber.

Device fabrication

Four-inch silicon wafers with 2 µm of silicon oxide were protected by photoresist (S1813, spun on and baked at 100 °C for 2 minutes) and diced into one-by-two-inch pieces. After dicing the pieces were cleaned with 1165 photoresist remover at 100°C for 10 minutes, rinsed with isopropyl alcohol (IPA) and dried with nitrogen. The chips were then cleaned before silicon nitride deposition. The first step is a Piranha clean with H_2SO_4:H_2O_2 at 60:1 ratio and at a temperature of 100 °C. The second step is an RCA SC-1 clean. The solution is NH_4OH: H_2O_2: H_2O at a 1:1:5 ratio and temperature of 75 °C with megasonic cleaning. The third step is an RCA SC-2 clean. The solution consists of HCl: H_2O_2: H_2O at a ratio of 1:1:5 and temperature of 75 °C with megasonic cleaning. Silicon nitride was then deposited on clean pieces using low pressure chemical vapor deposition with gas flow of 32 sccm dichlorosilane and 100 sccm ammonia with a temperature range of 827-837°C. After silicon nitride deposition, photoresist ZEP520A was spun on at 500 rpm for 10 seconds then 4000 rpm for 30 seconds and baked for 2 minutes at 180°C. The resonator pattern was then defined on the pieces using electron beam lithography (Elionix ELS 100). The EBL settings were a field size of 500,000 dots and 1000 µm. The writing order for fields was to go along the photonic circuit, so to pattern one resonator from coupler to coupler all at once. The feed pitch and scan pitch were both 5 dot. The beam current was 1 nA. The dose time was 0.240 µsec/dot, resulting in a dose of 240 µC/cm^2. The pieces were then developed with xylenes and IPA for 1 minutes and 30 seconds, respectively. After development, the pattern in the resist must be transferred to the silicon nitride. This transfer was done via plasma etch using a Plasma-Therm 790 reactive ion etcher. A mix of 45 sccm CF_4 and 5 sccm O_2

is used as the etch plasma. The power is set to 100 W and the pressure is set to 40 mT. The DC bias tends to vary between 240-250V. After etching any remaining electron beam resist is removed by soaking the pieces in 1165 remover at 110°C for 20 minutes. The pieces are then rinsed with IPA and dried with nitrogen.

Simulation details

The optical mode, Figure 3, was modeled using the "Axisymmetric Cavity Resonator" guide as implemented in COMSOL Multiphysics®.[12] Optical mode profile details: The overall simulation area was set to be a 5x5 µm box, the waveguide (275x450 nm) was centered at coordinates (0,0), the rest of the box was set as air on top of the waveguide and silicon oxide on the bottom. Refractive index for the silicon nitride was set at 2.02, silicon oxide at 1.44, and air at 1. Electromagnetic Waves, Frequency Domain module was used, the Wave Equation was applied to all domains, and Initial Values for electric field were set at 0. The Scattering Boundary Condition was applied to the box boundary. Mesh for the waveguide was set to a maximum element size of 5 nm. The rest of the model uses generic "Fine" size for the mesh. The resultant field was calculated by searching for eigenfrequencies around 780 µm or 384.3 THz. The data was exported as a text file, with pointes evaluated in "regular grid", data format as a "grid" and number of R and Z points both set to 3616. The result was imported as a "Interpolation" into the thermal simulation suite.

Thermal simulations were created using cylinder geometry to represent each layer of the platform. Silicon nitride MRR was built to have height of 275 nm, radius of 30, 45, or 60 µm and width of 450 nm. Silicon oxide layer was set at height of 2 µm, and silicon layer was set at height of 1000 µm. 200 µm tall layer of air was on top of the platform (Figure S1a). All the materials were imported from COMSOL Multiphysics library. "Heat Transfer in Solids" module

was used, initial temperature of all domains was set at 300 K, the temperature of all the outside boundaries was set to be constant at 300 K to account for thermal insulation. Mesh sizes were set at “normal” for silicon and air domains, and “extremely fine” for silicon nitride and silicon oxide. Both stationary and time dependent solutions were computed. The photothermal wavelength shift was calculated using “Volume Integration” between optical and thermal simulation results.

The thermo-optical simulation was build following the “Modeling the Pulsed Laser Heating of Semitransparent Materials” blog post was used as a guide.[13] The geometry used was the same as the geometry used for the single-particle thermal simulation. The absorption coefficient for silicon nitride and silicon oxide were set to 0, assuming those layers are transparent at 635 nm excitation wavelength, absorption coefficient of silicon was calculated to be 1979 1/m, assuming imaginary component of refractive index is 10^{-5}. The incident intensity was defined as input power in W to mimic the experiment, 500 µW. The beam radius was set at 350 nm. The position of the incident intensity was a varying parameter from -r-15 µm to r+15 µm with 500 nm step, where r is MRR radius. Stationary (steady state) solution was calculated for each incident intensity position.

Thermal Simulations of different platforms

Figure S1 shows the cross-section of the elevated temperature profile after photothermal pumping of an absorber on several potential materials geometries. Figure S1a shows the general layout: MRR (Pink), air (gray), thin layer (green), and substrate (blue). Figure S1b shows results for the platform investigated in our experiment: 45 µm-diameter silicon nitride MRR on 2 µm layer of silicon oxide on a 1000 µm layer of silicon, with 200 µm layer of air on top. Figure S1c

models a 45 µm- diameter MRR on 1002 µm layer of silicon oxide (no silicon). Figure S1d corresponds to a 45 µm-diameter MRR suspended in air (an unphysical geometry).

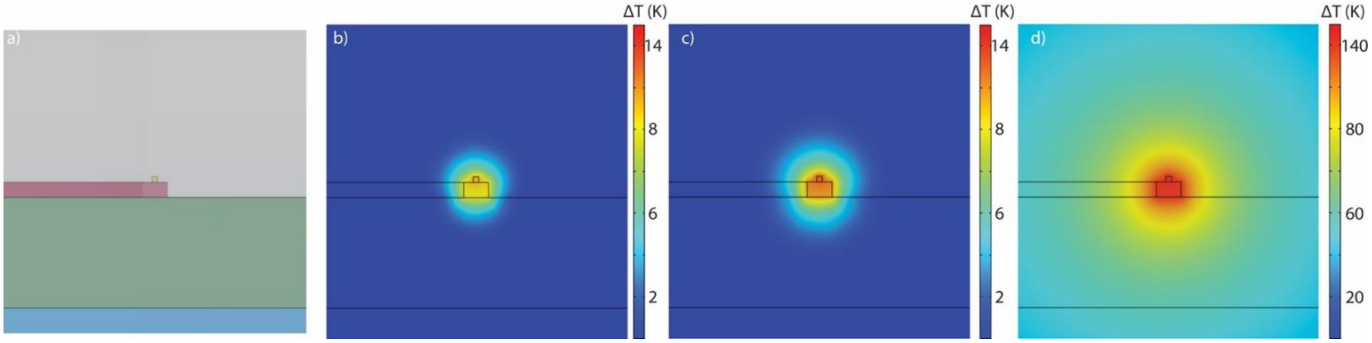

Figure S1. a) General simulation geometry. b) Cross section of temperature profile of silicon nitride-silicon oxide-silicon platform. c) Cross-section of temperature profile of silicon nitride-silicon oxide platform. d) Cross-section of temperature profile of a suspended silicon nitride in air platform. Note, the temperature scale of panel d) is larger than in b,c).

Optical characterization of MRRs

Three MRR designs were evaluated, 30, 45 or 60 μm diameter, Fig S2. 45 μm diameter was chosen for the final experiments due to the best Q/V ratio. The Q-factor goes down when any particles are deposited on top of MRRs, so 30 μm diameter MRRs were not suitable for photothermal experiments post particle deposition due to lower transmission and Q-factors only reaching $20x10^4$. The 60 μm diameter MRRs have lower overlap between optical mode and the single particle temperature profile.

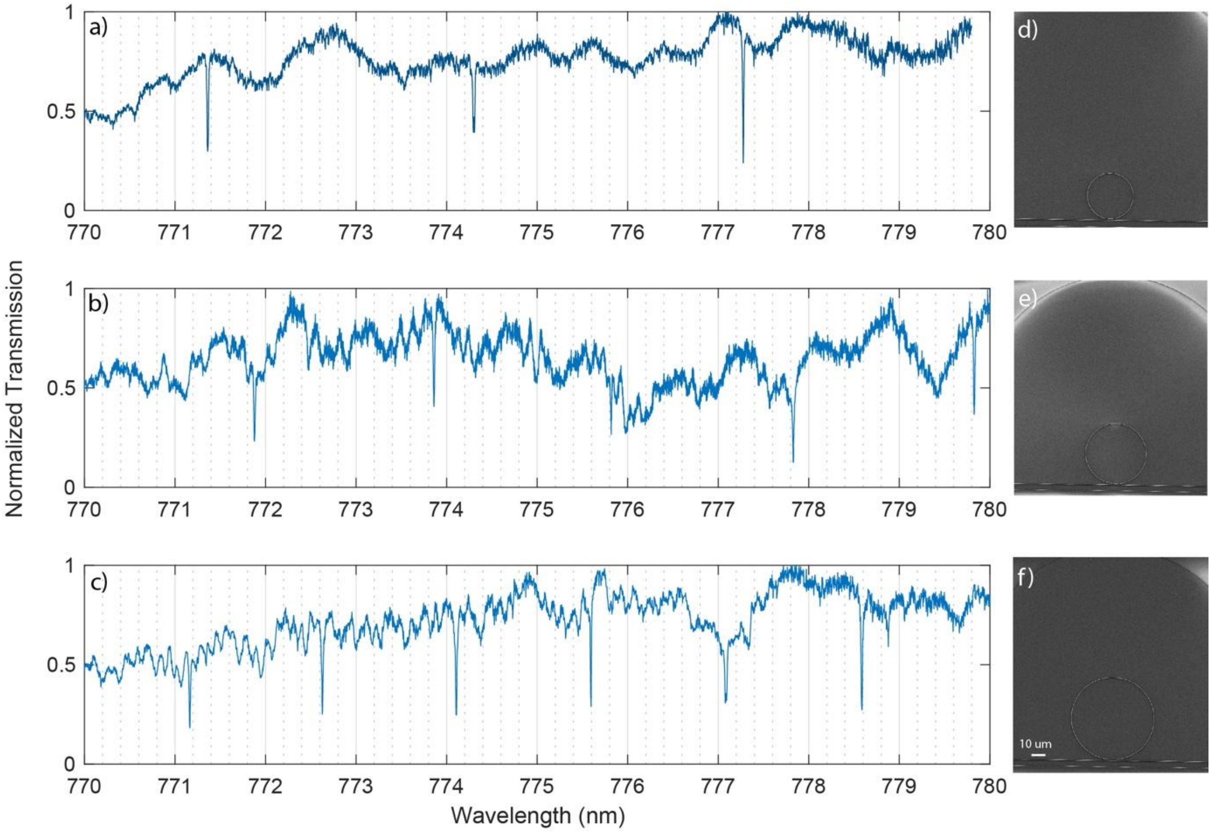

Figure S2. Optical transmission of 30, 40 and 60 μm diameter MRR post fabrication, before any particle depositions took place along with electron micrographs: a) and d), b) and e), c) and f) respectively.

Photothermal mapping of MWCNTs

Figure S3 shows the SEM images, photothermal maps and the overlap between the two images collected on the same MRR. MWCNTs can be seen as bright spots in the coarse and fine photothermal maps and correlate well with features observed in the SEM micrograph. When taking photothermal data we are able to see the MRR, so the resultant maps are known to cover the same area of interest. The overlays were created by scaling the SEM image using the scale bar to the photothermal maps pixels, which are of know size (2 μm for the overall map and 500 nm for the individual maps of the particles). The position of the overlays was determined by relying on the photothermal background signal which is the shape of the MRR.

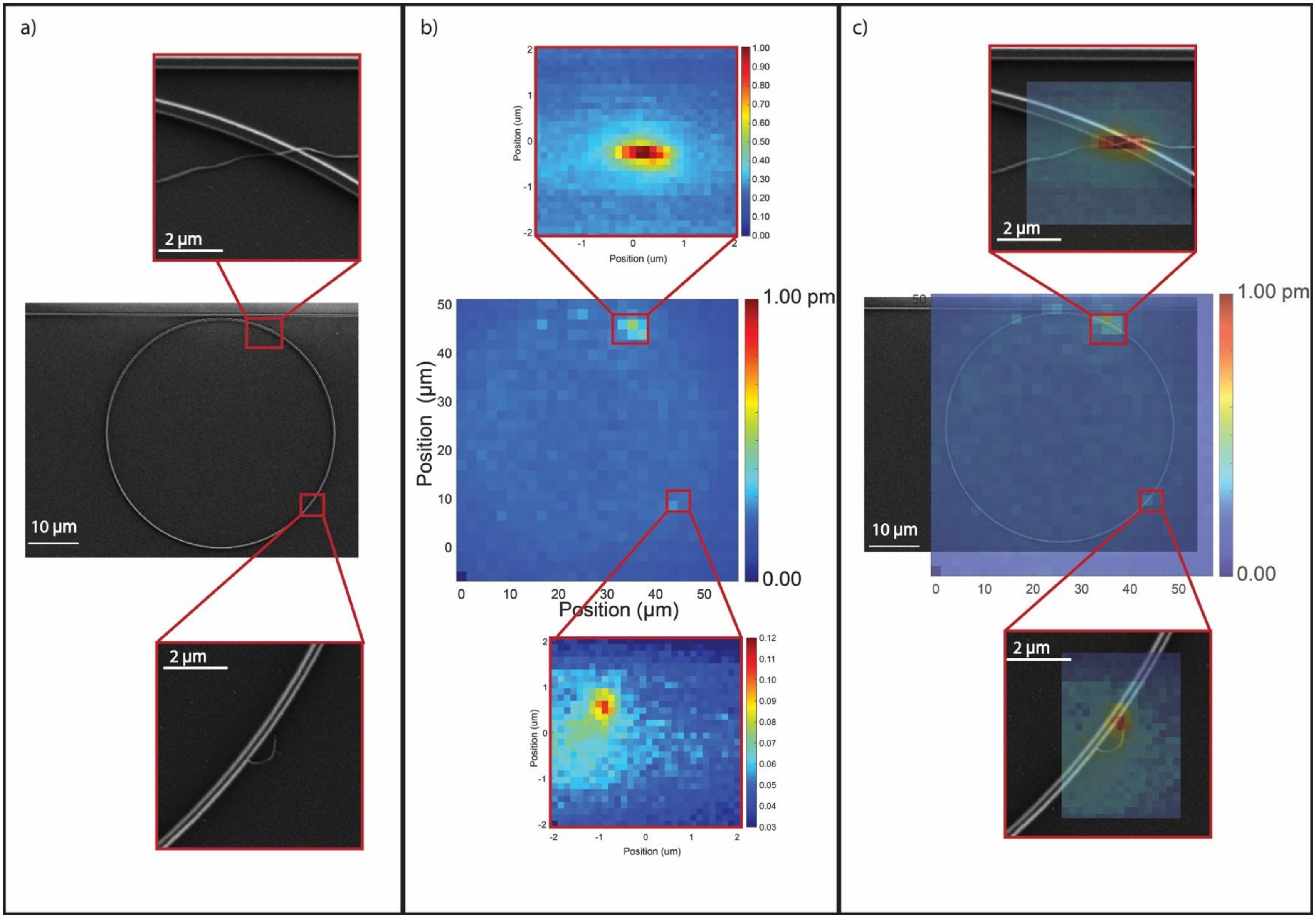

Figure S3. a) Scanning electron micrographs of a MRR with two distinct particles. b) Photothermal maps of the same areas collected with 635 nm excitation wavelength. c) Overlay of the SEM and photothermal maps.

References

(1) Aspnes, D. E.; Studna, A. A. Dielectric functions and optical parameters of Si, Ge, GaP, GaAs, GaSb, InP, InAs, and InSb from 1.5 to 6.0 eV. *Physical Review B* **1983**, *27* (2), 985-1009.
(2) Tan, C. Z. Determination of refractive index of silica glass for infrared wavelengths by IR spectroscopy. **1998**, *223* (1), 158-163.
(3) Luke, K.; Okawachi, Y.; Lamont, M. R. E.; Gaeta, A. L.; Lipson, M. Broadband mid-infrared frequency comb generation in a Si3N4 microresonator. *Opt. Lett.* **2015**, *40* (21), 4823-4826.
(4) Komma, J.; Schwarz, C.; Hofmann, G.; Heinert, D.; Nawrodt, R. Thermo-optic coefficient of silicon at 1550 nm and cryogenic temperatures. **2012**, *101* (4), 041905.
(5) Leviton, D. B.; Frey, B. J. Temperature-dependent Absolute Refractive Index Measurements of Synthetic Fused Silica. *SPIE Proceedings* **2006**, *6273*, 800-810.
(6) Arbabi, A.; Goddard, L. L. Measurements of the refractive indices and thermo-optic coefficients of Si3N4 and SiOx using microring resonances. *Opt. Lett.* **2013**, *38* (19), 3878-3881.
(7) Glassbrenner, C. J.; Slack, G. A. Thermal Conductivity of Silicon and Germanium from 3\ifmmode^\circ\else\textdegree\fi{}K to the Melting Point. *Physical Review* **1964**, *134* (4A), A1058-A1069.
(8) He, R.; Sun, B. Thermal conductivity of SiO2 grown by plasma enhanced chemical vapor deposition. **2025**, *137* (17), 175109.
(9) Pierson, H. O. *Handbook of chemical vapor deposition (CVD): principles, technology, and applications*; 1999.
(10) Marchetti, R.; Lacava, C.; Carroll, L.; Gradkowski, K.; Minzioni, P. Coupling strategies for silicon photonics integrated chips [Invited]. *Photon. Res.* **2019**, *7* (2), 201-239. DOI: 10.1364/PRJ.7.000201.
(11) Covey, J.; Chen, R. T. Efficient perfectly vertical fiber-to-chip grating coupler for silicon horizontal multiple slot waveguides. *Opt. Express* **2013**, *21* (9), 10886-10896. DOI: 10.1364/OE.21.010886.
(12) *Axisymmetric Cavity Resonator*. COMSOL Multiphysics®, https://www.comsol.com/model/axisymmetric-cavity-resonator-14517 (accessed 06/19/2024).
(13) Frei, W. Modeling the Pulsed Laser Heating of Semitransparent Materials. 2023; Vol. 2024.
